# Supplementary material for: High-fidelity endonuclease variant HypaCas9 facilitates accurate allele-specific gene modification in mouse zygotes
Source: Commun Biol. 2019 Oct 10;2:371. doi: 10.1038/s42003-019-0627-8 (PMC6787007; doi:10.1038/s42003-019-0627-8)
Supplement: Supplementary file 1 — Supplementary Information [file 42003_2019_627_MOESM1_ESM.pdf]

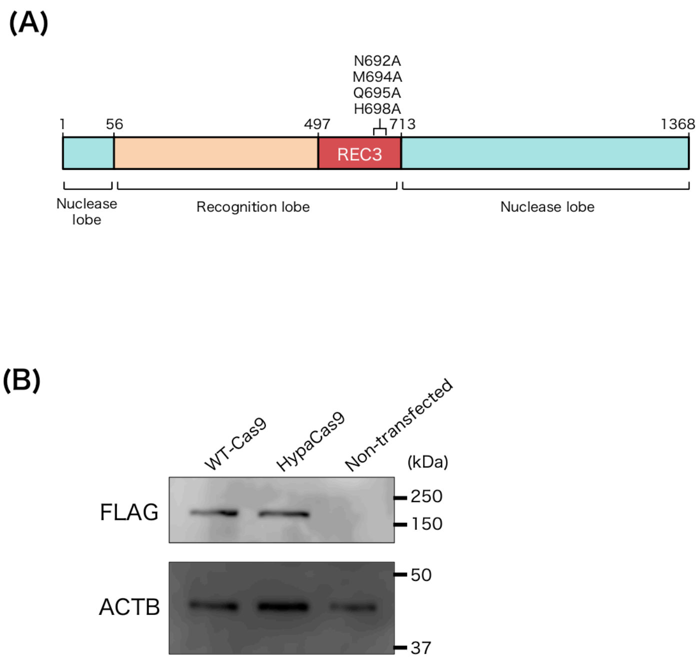

Supplementary Figure 1. Construction of the HypaCas9 expression plasmid.

(A) Schematic of HypaCas9 construction by alanine substitution. Four alanine substitutions were introduced into the REC3 domain. (B) Confirmation of HypaCas9 expression by immunoblotting. A band with the expected molecular weight (about 162 kDa) of HypaCas9 was observed in HEK293 cells. Beta-actin (ACTB) was used as a loading control.

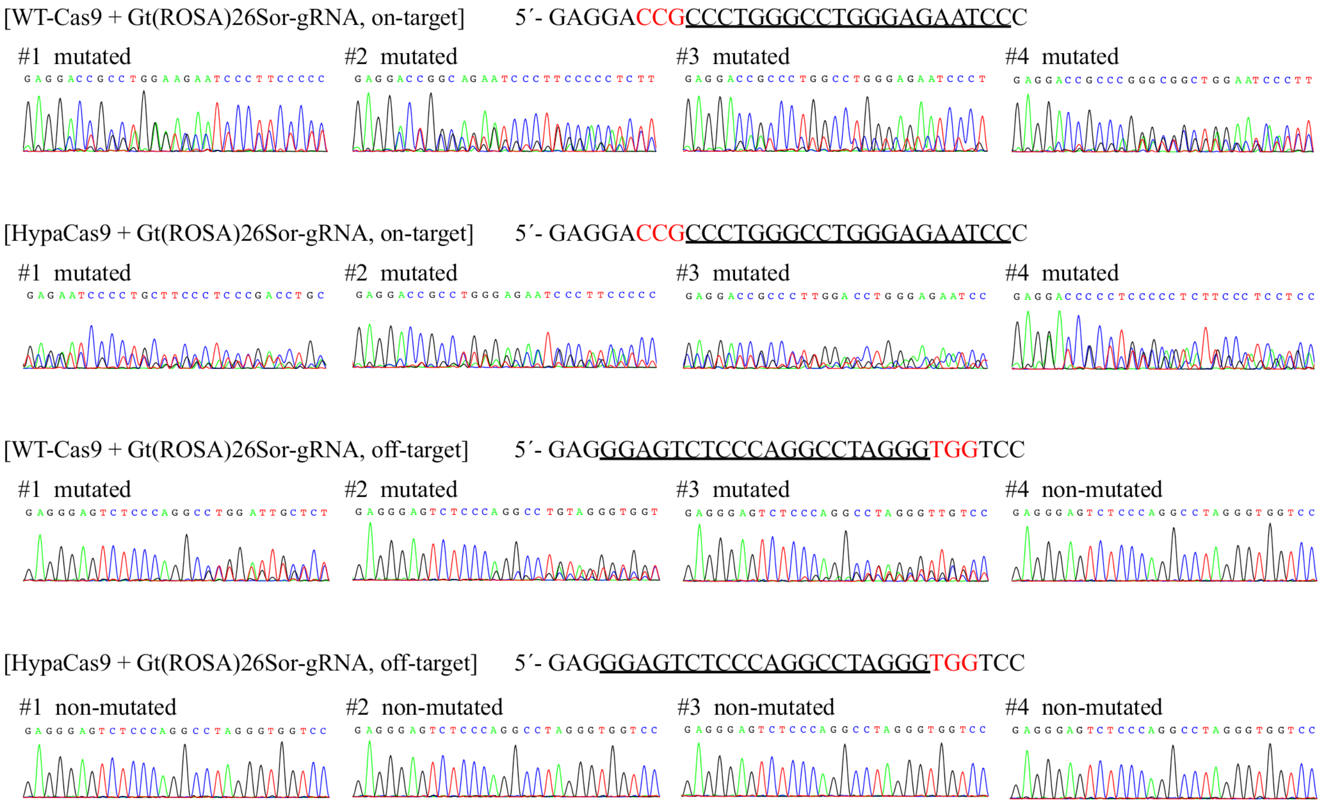

Supplementary Figure 2. Sequencing analysis in gene modification using *Gt(ROSA)26Sor*-gRNA.

Chromatogram data were obtained by Sanger sequencing in each embryo. Four representative results are shown in each experimental group. The wildtype sequences of the target site are also shown. The target sequence of gRNA is indicated by underlining and the PAM is shown in red.

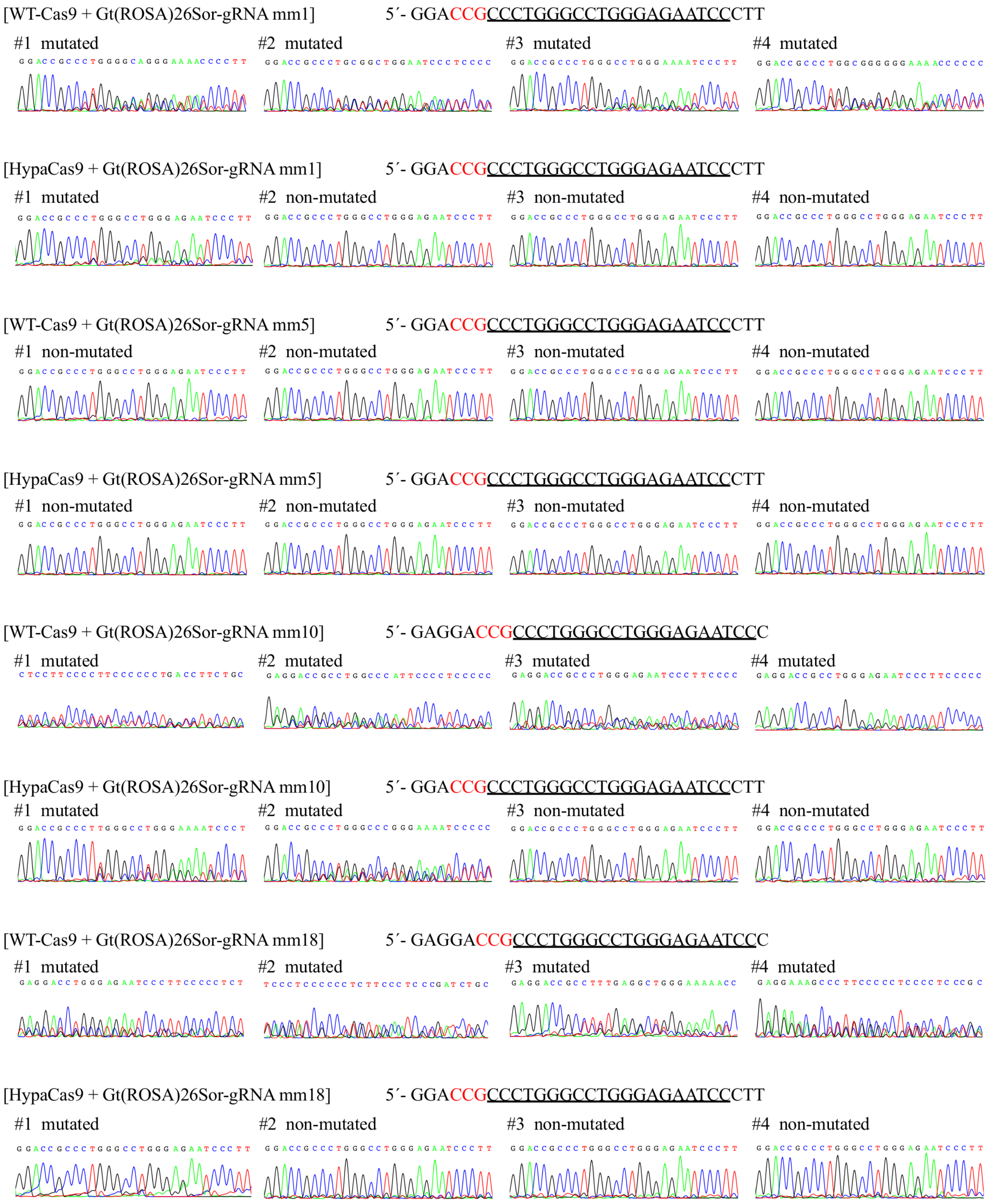

Supplementary Figure 3. Sequencing analysis in gene modification using *Gt(ROSA)26Sor*-gRNA with a single mismatch.

Chromatogram data obtained by Sanger sequencing in each embryo. Four representative results are shown in each experimental group. The wildtype sequences of the target site are also shown. The target sequence of gRNA is indicated by underlining and the PAM is shown in red.

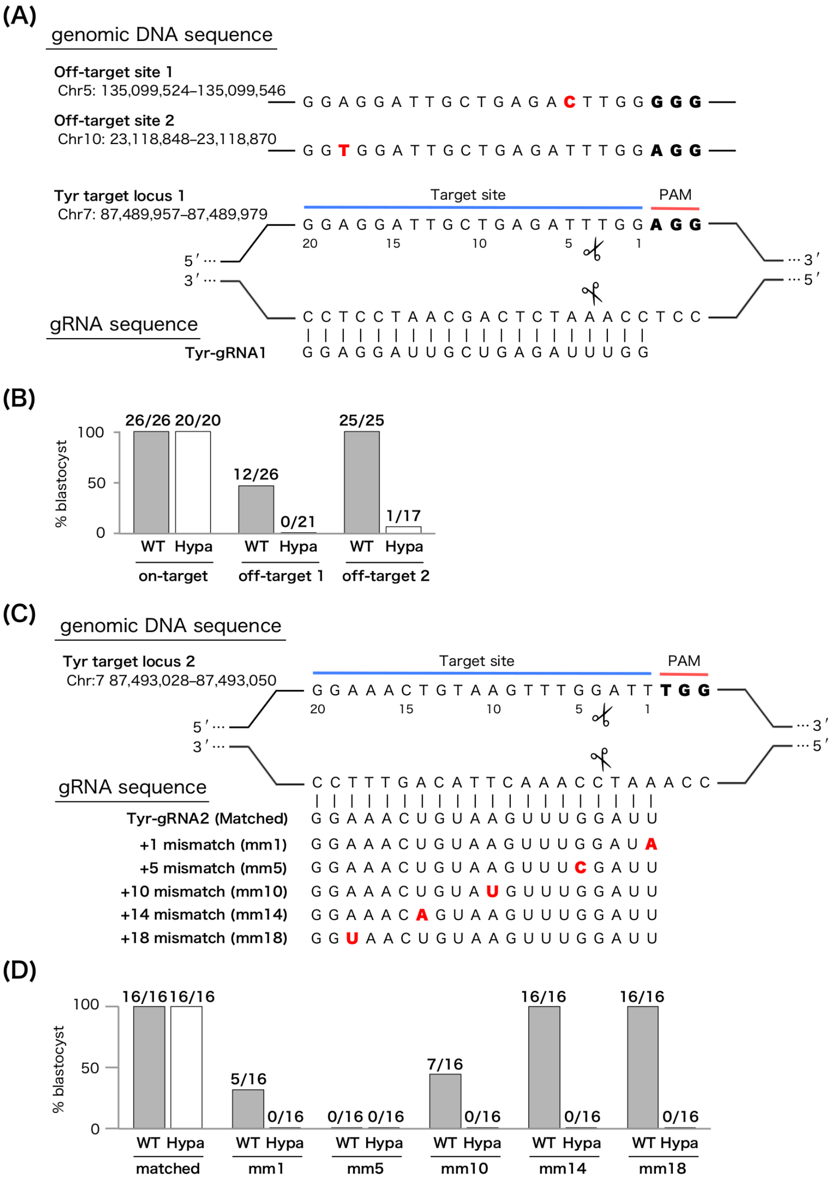

Supplementary Figure 4. Comparison of on- and off-target activity between WT-Cas9 and HypaCas9.

(A) Genomic DNA sequences of the *Tyr*-gRNA1 targeting site and two off-target sites. (B) Comparison of on- and off-target activity between WT-Cas9 and HypaCas9 using *Tyr*-gRNA1. (C) Genomic DNA sequence of the *Tyr*-gRNA2 targeting site, and the design of gRNAs with a single mismatch at position 1, 5, 10, 14, or 18. (D) Comparison of on- and off-target activity between WT-Cas9 and HypaCas9 using matched and singly mismatched *Tyr*-gRNA2. In (A and C), the mismatched nucleotides are shown in red, and the locus information is based on GRCm38.p4. In (B and D), the numbers above the bar indicate the numbers of mutated embryos per total embryos from two replicate experiments.

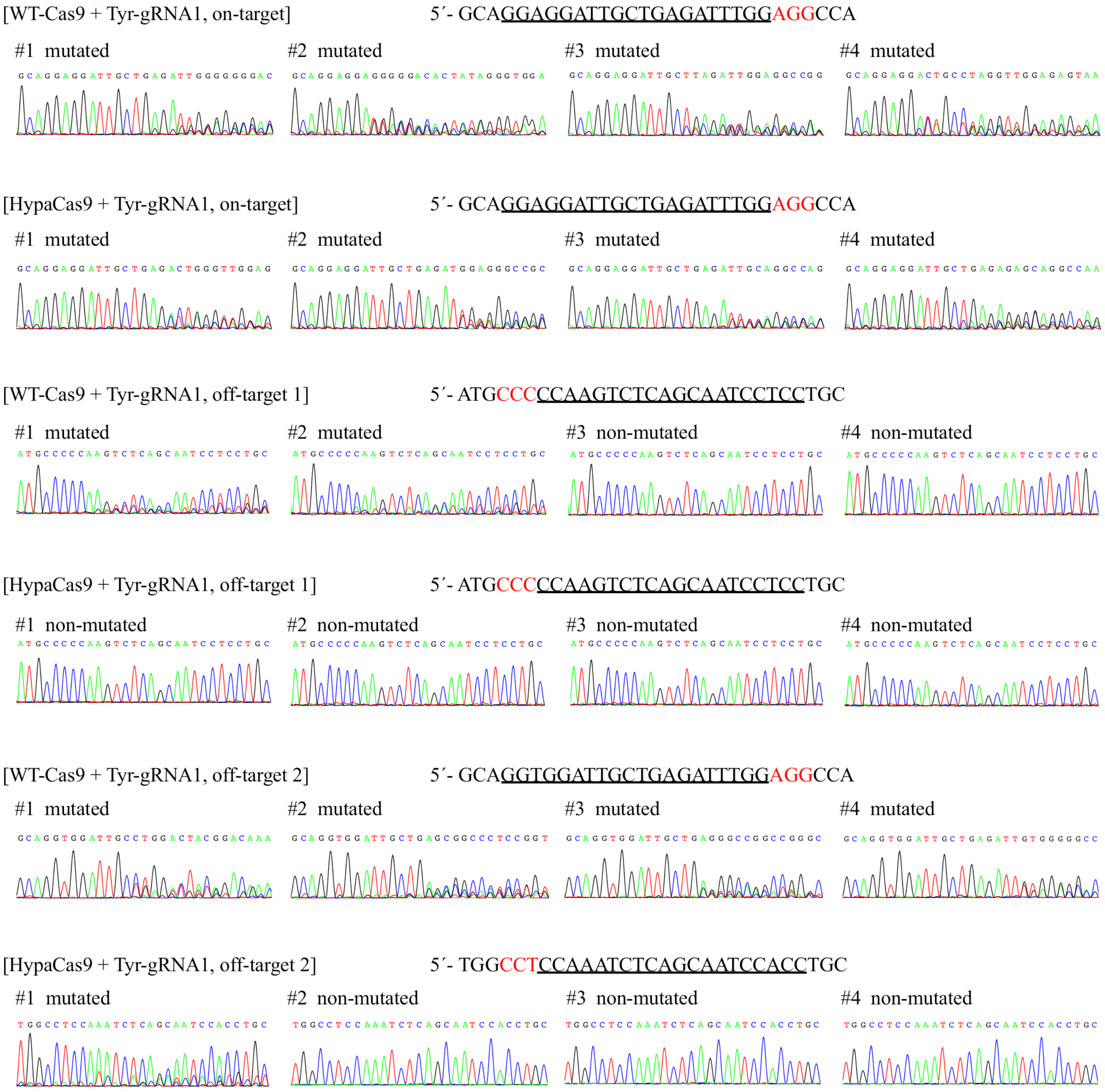

Supplementary Figure 5. Sequencing analysis in gene modification using *Tyr*-gRNA1.
Chromatogram data were obtained by Sanger sequencing in each embryo. Four representative results are shown in each experimental group. The wildtype sequences of the target site are also shown. The target sequence of gRNA is indicated by underlining and the PAM is shown in red.

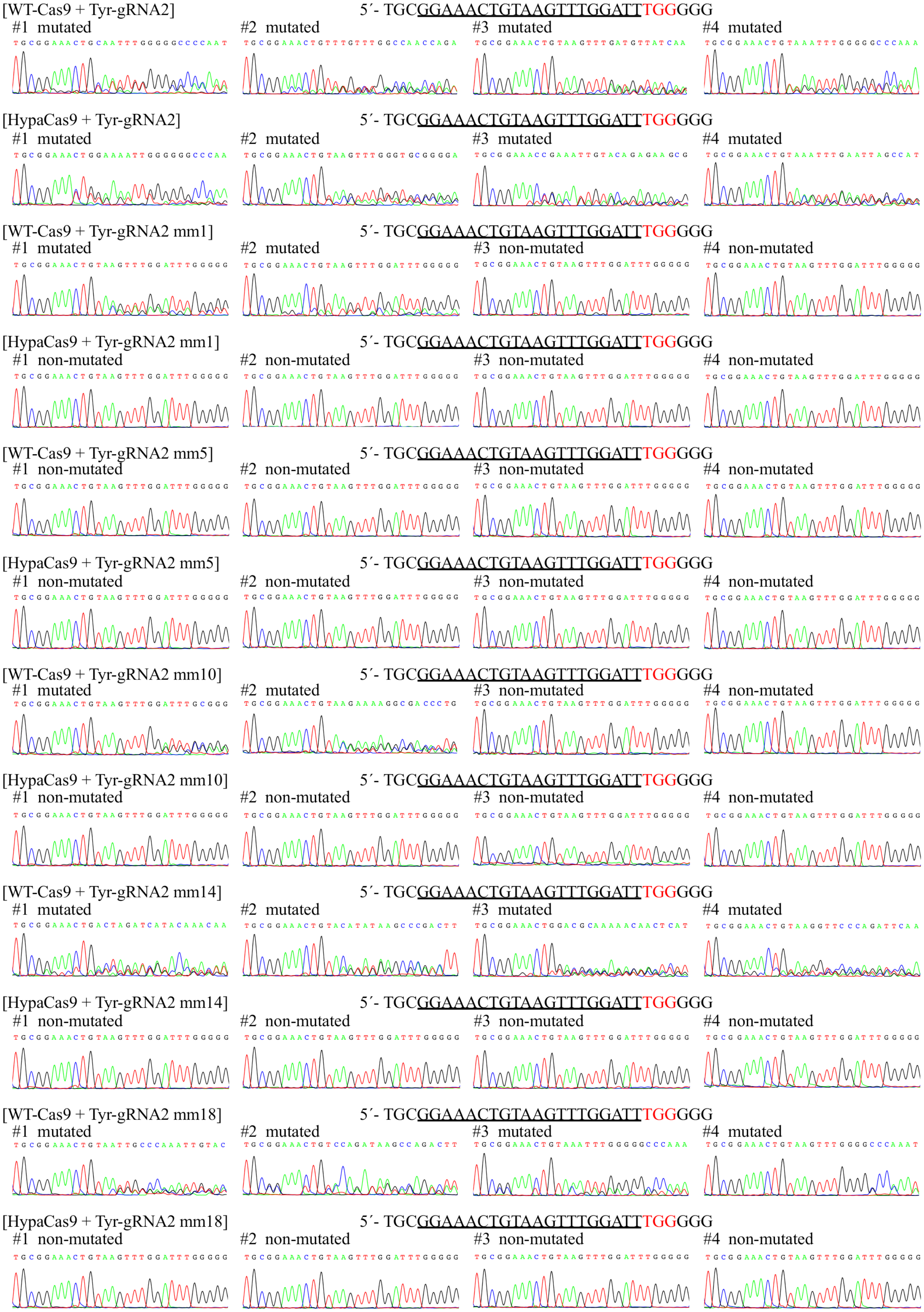

Supplementary Figure 6. Sequencing analysis in gene modification using *Tyr*-gRNA2.
Chromatogram data were obtained by Sanger sequencing in each embryo. Four representative results are shown in each experimental group. The wildtype sequences of the target site are also shown. The target sequence of gRNA is indicated by underlining and the PAM is shown in red.

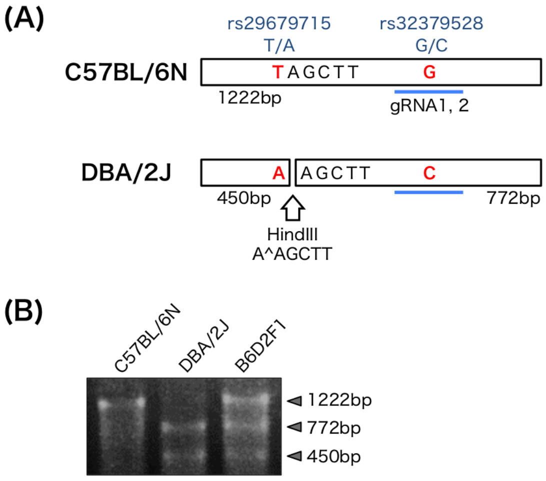

Supplementary Figure 7. Method for allele separation in B6D2F1 embryos.

(A) Schematic for separation of *Cdx2* PCR products from the C57BL/6N and DBA/2J alleles. SNP rs29679715 and rs32379528 are shown in red. The HindIII cut site is present only in the PCR product from the DBA/2J allele. (B) Electrophoretic pattern of *Cdx2* PCR products after digestion with HindIII. The C57BL/6N allele remains uncleaved (1222 bp) and the DBA/2J allele is cleaved (772 bp and 450 bp). Fragments of 1222 bp and 772 bp were sequenced independently for detection of allele-specific mutation.

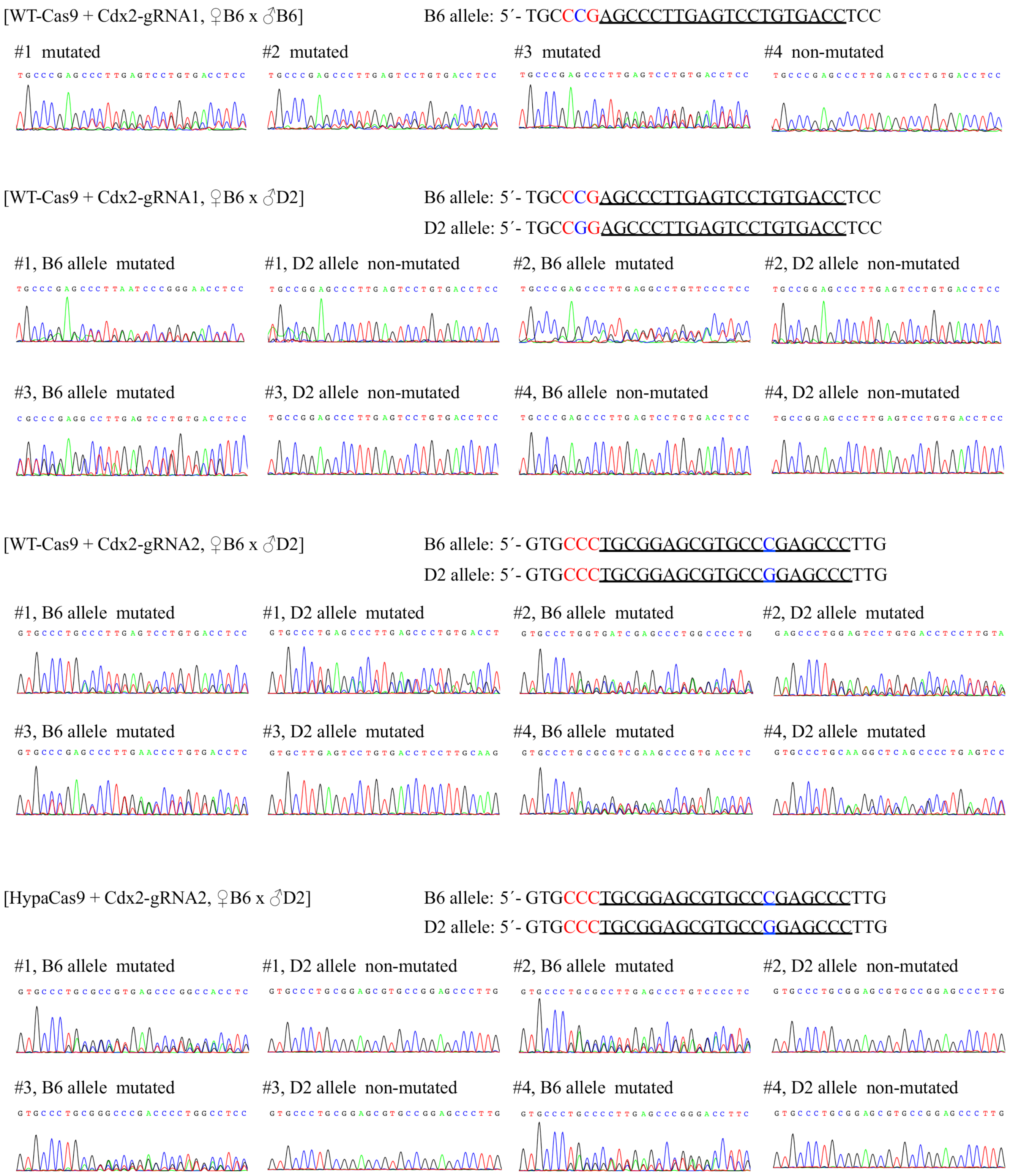

Supplementary Figure 8. Sequencing analysis in allele-specific gene modification targeting *Cdx2*.

Chromatogram data were obtained by Sanger sequencing in each embryo. Four representative results are shown in each experimental group. The wildtype sequences of the target site in C57BL/6N (B6) and DBA/2J (D2) allele are also shown. The target sequence of gRNA is indicated by underlining, the PAM is shown in red, and SNP rs32379528 is shown in blue.

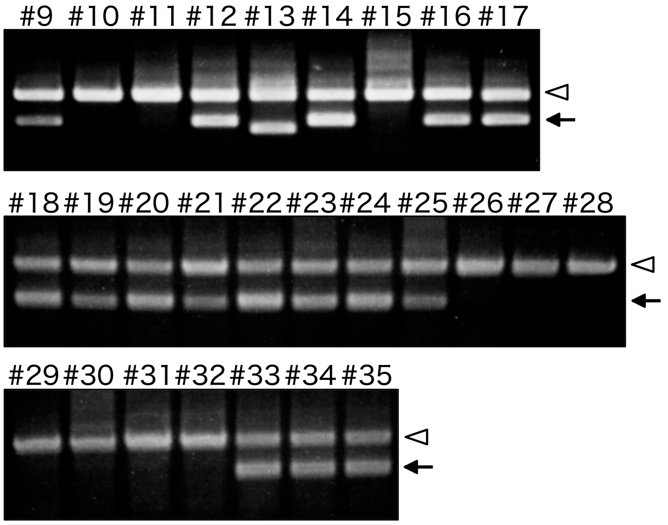

Supplementary Figure 9. Monoallelic genome modification by HypaCas9.
PCR analysis of the monoallelic deletion of *Cdk1* exon 3 in F0 mice Nos. 9–35. The white arrowhead indicates the WT allele or an allele with a small indel. The arrow indicates the deletion allele.

Supplementary Figure 10. Sequences of HypaCas9 and gRNAs.

In the DNA sequence of the HypaCas9 vector and amino acid sequence of HypaCas9, the start and stop codons are underlined, T3 promoter is shown in green, Flag-tag in red, NLS in orange, and HypaCas9 in blue. In addition, four alanine substitutions are indicated by yellow lines.

>DNA sequence of HypaCas9 vector (from T3 promoter to SphI site)

GA**AATTAACCCTCACTAAAGG**GAACAAAAGCTGGAGCTCCACCGCGGTGGCGGCCGC
TCTAGCCCGGGCGGATCCACC**ATG****GATTACAAGGATGACGACGATAAG**ATCATGGCC
CCAAAGAAGAAGCGGAAGGTCGGTATCCACGGAGTCCCAGCAGCC**GACAAGAAGTA
CTCCATTGGGCTCGATATCGGCACAAACAGCGTCGGCTGGGCCGTCATTACGGAC
GAGTACAAGGTGCCGAGCAAAAAATTCAAAGTTCTGGGCAATACCGATCGCCACA
GCATAAAGAAGAACCTCATTGGCGCCCTCCTGTTCGACTCCGGGGAGACGGCCGA
AGCCACGCGGCTCAAAAGAACAGCACGGCGCAGATATACCCGCAGAAAGAATCGG
ATCTGCTACCTGCAGGAGATCTTTAGTAATGAGATGGCTAAGGTGGATGACTCTTT
CTTCCATAGGCTGGAGGAGTCCTTTTTGGTGGAGGAGGATAAAAAGCACGAGCGC
CACCCAATCTTTGGCAATATCGTGGACGAGGTGGCGTACCATGAAAAGTACCCAA
CCATATATCATCTGAGGAAGAAGCTTGTAGACAGTACTGATAAGGCTGACTTGCG
GTTGATCTATCTCGCGCTGGCGCATATGATCAAATTTCGGGGACACTTCCTCATCG
AGGGGGACCTGAACCCAGACAACAGCGATGTCGACAAACTCTTTATCCAACTGGT
TCAGACTTACAATCAGCTTTTCGAAGAGAACCCGATCAACGCATCCGGAGTTGACG
CCAAAGCAATCCTGAGCGCTAGGCTGTCCAAATCCCGGCGGCTCGAAAACCTCAT
CGCACAGCTCCCTGGGGAGAAGAAGAACGGCCTGTTTGGTAATCTTATCGCCCTG
TCACTCGGGCTGACCCCCAACTTTAAATCTAACTTCGACCTGGCCGAAGATGCCAA
GCTTCAACTGAGCAAAGACACCTACGATGATGATCTCGACAATCTGCTGGCCCAG
ATCGGCGACCAGTACGCAGACCTTTTTTTGGCGGCAAAGAACCTGTCAGACGCCA
TTCTGCTGAGTGATATTCTGCGAGTGAACACGGAGATCACCAAAGCTCCGCTGAG
CGCTAGTATGATCAAGCGCTATGATGAGCACCACCAAGACTTGACTTTGCTGAAG
GCCCTTGTCAGACAGCAACTGCCTGAGAAGTACAAGGAAATTTTCTTCGATCAGTC
TAAAAATGGCTACGCCGGATACATTGACGGCGGAGCAAGCCAGGAGGAATTTTAC
AAATTTATTAAGCCCATCTTGGAAAAAATGGACGGCACCGAGGAGCTGCTGGTAA
AGCTTAACAGAGAAGATCTGTTGCGCAAACAGCGCACTTTCGACAATGGAAGCAT
CCCCCACCAGATTCACCTGGGCGAACTGCACGCTATCCTCAGGCGGCAAGAGGAT
TTCTACCCCTTTTTGAAAGATAACAGGGAAAAGATTGAGAAAATCCTCACATTTCG
GATACCCTACTATGTAGGCCCCCTCGCCCGGGGAAATTCCAGATTCGCGTGGATG
ACTCGCAAATCAGAAGAGACCATCACTCCCTGGAACTTCGAGGAAGTCGTGGATA
AGGGGGCCTCTGCCCAGTCCTTCATCGAAAGGATGACTAACTTTGATAAAAATCTG**

CCTAACGAAAAGGTGCTTCCTAAACACTCTCTGCTGTACGAGTACTTCACAGTTTA

TAACGAGCTCACCAAGGTCAAATACGTCACAGAAGGGATGAGAAAGCCAGCATTC

CTGTCTGGAGAGCAGAAGAAAGCTATCGTGGACCTCCTCTTCAAGACGAACCGGA

AAGTTACCGTGAAACAGCTCAAAGAAGACTATTTCAAAAAGATTGAATGTTTCGAC

TCTGTTGAAATCAGCGGAGTGGAGGATCGCTTCAACGCATCCCTGGGAACGTATC

ACGATCTCCTGAAAATCATTAAAGACAAGGACTTCCTGGACAATGAGGAGAACGA

GGACATTCTTGAGGACATTGTCCTCACCCTTACGTTGTTTGAAGATAGGGAGATGA

TTGAAGAACGCTTGAAAACTTACGCTCATCTCTTCGACGACAAAGTCATGAAACAG

CTCAAGAGGCGCCGATATACAGGATGGGGGCGGCTGTCAAGAAAACTGATCAATG

GGATCCGAGACAAGCAGAGTGGAAAGACAATCCTGGATTTTCTTAAGTCCGATGG

ATTTGCCAACCGGgccTTCgctgcaTTGATCgctGATGACTCTCTCACCTTTAAGGAGGA

CATCCAGAAAGCACAAGTTTCTGGCCAGGGGGACAGTCTTCACGAGCACATCGCT

AATCTTGCAGGTAGCCCAGCTATCAAAAAGGGAATACTGCAGACCGTTAAGGTCG

TGGATGAACTCGTCAAAGTAATGGGAAGGCATAAGCCCGAGAATATCGTTATCGA

GATGGCCCGAGAGAACCAAACTACCCAGAAGGGACAGAAGAACAGTAGGGAAAG

GATGAAGAGGATTGAAGAGGGTATAAAAGAACTGGGGTCCCAAATCCTTAAGGAA

CACCCAGTTGAAAACACCCAGCTTCAGAATGAGAAGCTCTACCTGTACTACCTGCA

GAACGGCAGGGACATGTACGTGGATCAGGAACTGGACATCAATCGGCTCTCCGAC

TACGACGTGGATCATATCGTGCCCCAGTCTTTTCTCAAAGATGATTCTATTGATAA

TAAAGTGTTGACAAGATCCGATAAAAATAGAGGGAAGAGTGATAACGTCCCCTCA

GAAGAAGTTGTCAAGAAAATGAAAAATTATTGGCGGCAGCTGCTGAACGCCAAAC

TGATCACACAACGGAAGTTCGATAATCTGACTAAGGCTGAACGAGGTGGCCTGTC

TGAGTTGGATAAAGCCGGCTTCATCAAAAGGCAGCTTGTTGAGACACGCCAGATC

ACCAAGCACGTGGCCCAAATTCTCGATTCACGCATGAACACCAAGTACGATGAAA

ATGACAAACTGATTCGAGAGGTGAAAGTTATTACTCTGAAGTCTAAGCTGGTCTCA

GATTTCAGAAAGGACTTTCAGTTTTATAAGGTGAGAGAGATCAACAATTACCACCA

TGCGCATGATGCCTACCTGAATGCAGTGGTAGGCACTGCACTTATCAAAAAATATC

CCAAGCTTGAATCTGAATTTGTTTACGGAGACTATAAAGTGTACGATGTTAGGAAA

ATGATCGCAAAGTCTGAGCAGGAAATAGGCAAGGCCACCGCTAAGTACTTCTTTTA

CAGCAATATTATGAATTTTTTCAAGACCGAGATTACACTGGCCAATGGAGAGATTC

GGAAGCGACCACTTATCGAAACAAACGGAGAAACAGGAGAAATCGTGTGGGACAA

GGGTAGGGATTTCGCGACAGTCCGGAAGGTCCTGTCCATGCCGCAGGTGAACATC

GTTAAAAAGACCGAAGTACAGACCGGAGGCTTCTCCAAGGAAAGTATCCTCCCGA

AAAGGAACAGCGACAAGCTGATCGCACGCAAAAAAGATTGGGACCCCAAGAAATA

CGGCGGATTCGATTCTCCTACAGTCGCTTACAGTGTACTGGTTGTGGCCAAAGTG

GAGAAAGGGAAGTCTAAAAAACTCAAAAGCGTCAAGGAACTGCTGGGCATCACAA

TCATGGAGCGATCAAGCTTCGAAAAAAACCCCATCGACTTTCTCGAGGCGAAAGG

ATATAAAGAGGTCAAAAAAGACCTCATCATTAAGCTTCCCAAGTACTCTCTCTTTG

AGCTTGAAAACGGCCGGAAACGAATGCTCGCTAGTGCGGGCGAGCTGCAGAAGG
TAACGAGCTGGCACTGCCCTCTAAATACGTTAATTTCTTGTATCTGGCCAGCCACT
ATGAAAAGCTCAAAGGGTCTCCCGAAGATAATGAGCAGAAGCAGCTGTTCGTGGA
ACAACACAAACACTACCTTGATGAGATCATCGAGCAAATAAGCGAATTCTCCAAAA
GAGTGATCCTCGCCGACGCTAACCTCGATAAGGTGCTTTCTGCTTACAATAAGCAC
AGGGATAAGCCCATCAGGGAGCAGGCAGAAAACATTATCCACTTGTTTACTCTGA
CCAACTTGGGCGCGCCTGCAGCCTTCAAGTACTTCGACACCACCATAGACAGAAA
GCGGTACACCTCTACAAAGGAGGTCCTGGACGCCACACTGATTCATCAGTCAATTA
CGGGGCTCTATGAAACAAGAATCGACCTCTCTCAGCTCGGTGGAGACAGCAGGGCT
GAC**CCCAAGAAGAAGAGGAAGGTG**__**TGA**__ATCGATGGTACCTATGGGCACCAAAGAAC
CTGTAAACGTTATCTTTTTAAATTGAATGTGCACAAATAAAAGTTTGGAAAAGAAAAAA
AAAAAAAAAAAAAAAAAAAAAAAAAAAAAAAAAAAAAAAAAAAAAAAAAAAAAAAA
AAAAAAAAAAAAAAAAAAAAAAAAAAAAAAAAACCCTCGAGGTAGCATGC

>Amino acid sequence of HypaCas9
MDYKDDDDKIMAPKKKRKVGIHGVPAADKKYSIGLDIGTNSVGWAVITDEYKVPSKKFKV
LGNTDRHSIKKNLIGALLFDSGETAEATRLKRTARRRYTRRKNRICYLQEIFSNEMAKVDDS
FFHRLEESFLVEEDKKHERHPIFGNIVDEVAYHEKYPTIYHLRKKLVDSTDKADLRLIYLAL
AHMIKFRGHFLIEGDLNPDNSDVDKLFIQLVQTYNQLFEENPINASGVDAKAILSARLSKSRR
LENLIAQLPGEKKNGLFGNLIALSLGLTPNFKSNFDLAEDAKLQLSKDTYDDDLDNLLAQIG
DQYADLFLAAKNLSDAILLSDILRVNTEITKAPLSASMIKRYDEHHQDLTLLKALVRQQLPE
KYKEIFFDQSKNGYAGYIDGGASQEEFYKFIKPILEKMDGTEELLVKLNREDLLRKQRTFDN
GSIPHQIHLGELHAILRRQEDFYPFLKDNREKIEKILTFRIPYYVGPLARGNSRFAWMTRKSE
ETITPWNFEEVVDKGASAQSFIERMTNFDKNLPNEKVLPKHSLLYEYFTVYNELTKVKYVT
EGMRKPAFLSGEQKKAIVDLLFKTNRKVTVKQLKEDYFKKIECFDSVEISGVEDRFNASLGT
YHDLLKIIKDKDFLDNEENEDILEDIVLTLTLFEDREMIEERLKTYAHLFDDKVMKQLKRRR
YTGWGRLSRKLINGIRDKQSGKTILDFLKSDGFANRAFAALIADDSLTFKEDIQKAQVSGQG
DSLHEHIANLAGSPAIKKGILQTVKVVDELVKVMGRHKPENIVIEMARENQTTQKGQKNSR
ERMKRIEEGIKELGSQILKEHPVENTQLQNEKLYLYYLQNGRDMYVDQELDINRLSDYDVD
HIVPQSFLKDDSIDNKVLTRSDKNRGKSDNVPSEEVVKKMKNYWRQLLNAKLITQRKFDNL
TKAERGGLSELDKAGFIKRQLVETRQITKHVAQILDSRMNTKYDENDKLIREVKVITLKSKL
VSDFRKDFQFYKVREINNYHHAHDAYLNAVVGTALIKKYPKLESEFVYGDYKVYDVRKMI
AKSEQEIGKATAKYFFYSNIMNFFKTEITLANGEIRKRPLIETNGETGEIVWDKGRDFATVRK
VLSMPQVNIVKKTEVQTGGFSKESILPKRNSDKLIARKKDWDPKKYGGFDSPTVAYSVLVV
AKVEKGKSKKLKSVKELLGITIMERSSFEKNPIDFLEAKGYKEVKKDLIIKLPKYSLFELENG
RKRMLASAGELQKGNELALPSKYVNFLYLASHYEKLKGSPEDNEQKQLFVEQHKHYLDEII
EQISEFSKRVILADANLDKVLSAYNKHRDKPIREQAENIIHLFTLTNLGAPAAFKYFDTTIDR
KRYTSTKEVLDATLIHQSITGLYETRIDLSQLGGDSRADPKKKRKV

In gRNA sequences, T3 promoter is shown in green, and template for the protospacer of gRNA is underlined.

>gRNA *Gt(ROSA)26Sor* (from T3 promoter to DraI site)
GA**AATTAACCCTCACTAAAGG**GGATTCTCCCAGGCCCAGGGGTTTTAGAGCTAGAAAT
AGCAAGTTAAAATAAGGCTAGTCCGTTATCAACTTGAAAAAGTGGCACCGAGTCGGTG
CTTTTAAA

>gRNA *Gt(ROSA)26Sor* mm1 (from T3 promoter to DraI site)
GA**AATTAACCCTCACTAAAGG**GGATTCTCCCAGGCCCAGGCGTTTTAGAGCTAGAAAT
AGCAAGTTAAAATAAGGCTAGTCCGTTATCAACTTGAAAAAGTGGCACCGAGTCGGTG
CTTTTAAA

>gRNA *Gt(ROSA)26Sor* mm5 (from T3 promoter to DraI site)
GA**AATTAACCCTCACTAAAGG**GGATTCTCCCAGGCCGAGGGGTTTTAGAGCTAGAAAT
AGCAAGTTAAAATAAGGCTAGTCCGTTATCAACTTGAAAAAGTGGCACCGAGTCGGTG
CTTTTAAA

>gRNA *Gt(ROSA)26Sor* mm10 (from T3 promoter to DraI site)
GA**AATTAACCCTCACTAAAGG**GGATTCTCCCTGGCCCAGGGGTTTTAGAGCTAGAAAT
AGCAAGTTAAAATAAGGCTAGTCCGTTATCAACTTGAAAAAGTGGCACCGAGTCGGTG
CTTTTAAA

>gRNA *Gt(ROSA)26Sor* mm18 (from T3 promoter to DraI site)
GA**AATTAACCCTCACTAAAGG**GGTTTCTCCCAGGCCCAGGGGTTTTAGAGCTAGAAAT
AGCAAGTTAAAATAAGGCTAGTCCGTTATCAACTTGAAAAAGTGGCACCGAGTCGGTG
CTTTTAAA

>gRNA1 *Tyr* (from T3 promoter to DraI site)
GA**AATTAACCCTCACTAAAGG**GGAGGATTGCTGAGATTTGGGTTTTAGAGCTAGAAAT
AGCAAGTTAAAATAAGGCTAGTCCGTTATCAACTTGAAAAAGTGGCACCGAGTCGGTG
CTTTTAAA

>gRNA2 *Tyr* (from T3 promoter to DraI site)
GA**AATTAACCCTCACTAAAGG**GGAAACTGTAAGTTTGGATTGTTTTAGAGCTAGAAAT
AGCAAGTTAAAATAAGGCTAGTCCGTTATCAACTTGAAAAAGTGGCACCGAGTCGGTG
CTTTTAAA

>gRNA2 *Tyr* mm1 (from T3 promoter to DraI site)
GA**AATTAACCCTCACTAAAGG**GGAAACTGTAAGTTTGGATAGTTTTAGAGCTAGAAAT
AGCAAGTTAAAATAAGGCTAGTCCGTTATCAACTTGAAAAAGTGGCACCGAGTCGGTG
CTTTTAAA

>gRNA2 *Tyr* mm5 (from T3 promoter to DraI site)
GA**AATTAACCCTCACTAAAGG**GGAAACTGTAAGTTTCGATTGTTTTAGAGCTAGAAAT
AGCAAGTTAAAATAAGGCTAGTCCGTTATCAACTTGAAAAAGTGGCACCGAGTCGGTG
CTTTTAAA

>gRNA2 *Tyr* mm10 (from T3 promoter to DraI site)
GA**AATTAACCCTCACTAAAGG**GGAAACTGTATGTTTGGATTGTTTTAGAGCTAGAAAT
AGCAAGTTAAAATAAGGCTAGTCCGTTATCAACTTGAAAAAGTGGCACCGAGTCGGTG
CTTTTAAA

>gRNA2 *Tyr* mm14 (from T3 promoter to DraI site)
GA**AATTAACCCTCACTAAAGG**GGAAACAGTAAGTTTGGATTGTTTTAGAGCTAGAAAT
AGCAAGTTAAAATAAGGCTAGTCCGTTATCAACTTGAAAAAGTGGCACCGAGTCGGTG
CTTTTAAA

>gRNA2 *Tyr* mm18 (from T3 promoter to DraI site)
GA**AATTAACCCTCACTAAAGG**GGTAACTGTAAGTTTGGATTGTTTTAGAGCTAGAAAT
AGCAAGTTAAAATAAGGCTAGTCCGTTATCAACTTGAAAAAGTGGCACCGAGTCGGTG
CTTTTAAA

>gRNA1 *Cdx2* (from T3 promoter to DraI site)
GA**AATTAACCCTCACTAAAGG**GGTCACAGGACTCAAGGGCTGTTTTAGAGCTAGAAAT
AGCAAGTTAAAATAAGGCTAGTCCGTTATCAACTTGAAAAAGTGGCACCGAGTCGGTG
CTTTTAAA

>gRNA2 *Cdx2* (from T3 promoter to DraI site)
GA**AATTAACCCTCACTAAAGG**GGGCTCGGGCACGCTCCGCAGTTTTAGAGCTAGAAAT
AGCAAGTTAAAATAAGGCTAGTCCGTTATCAACTTGAAAAAGTGGCACCGAGTCGGTG
CTTTTAAA

>gRNA *Cdk1* intron2 (from T3 promoter to DraI site)

GA**AATTAACCCTCACTAAAGG**GGGGCCTATCTGACTGCAGCGTTTTAGAGCTAGAAAT
AGCAAGTTAAAATAAGGCTAGTCCGTTATCAACTTGAAAAAGTGGCACCGAGTCGGTG
CTTTTAAA

>gRNA *Cdk1* intron3 (from T3 promoter to DraI site)
GA**AATTAACCCTCACTAAAGG**GGAGCTAAGCAAGCTCCCCAGTTTTAGAGCTAGAAAT
AGCAAGTTAAAATAAGGCTAGTCCGTTATCAACTTGAAAAAGTGGCACCGAGTCGGTG
CTTTTAAA

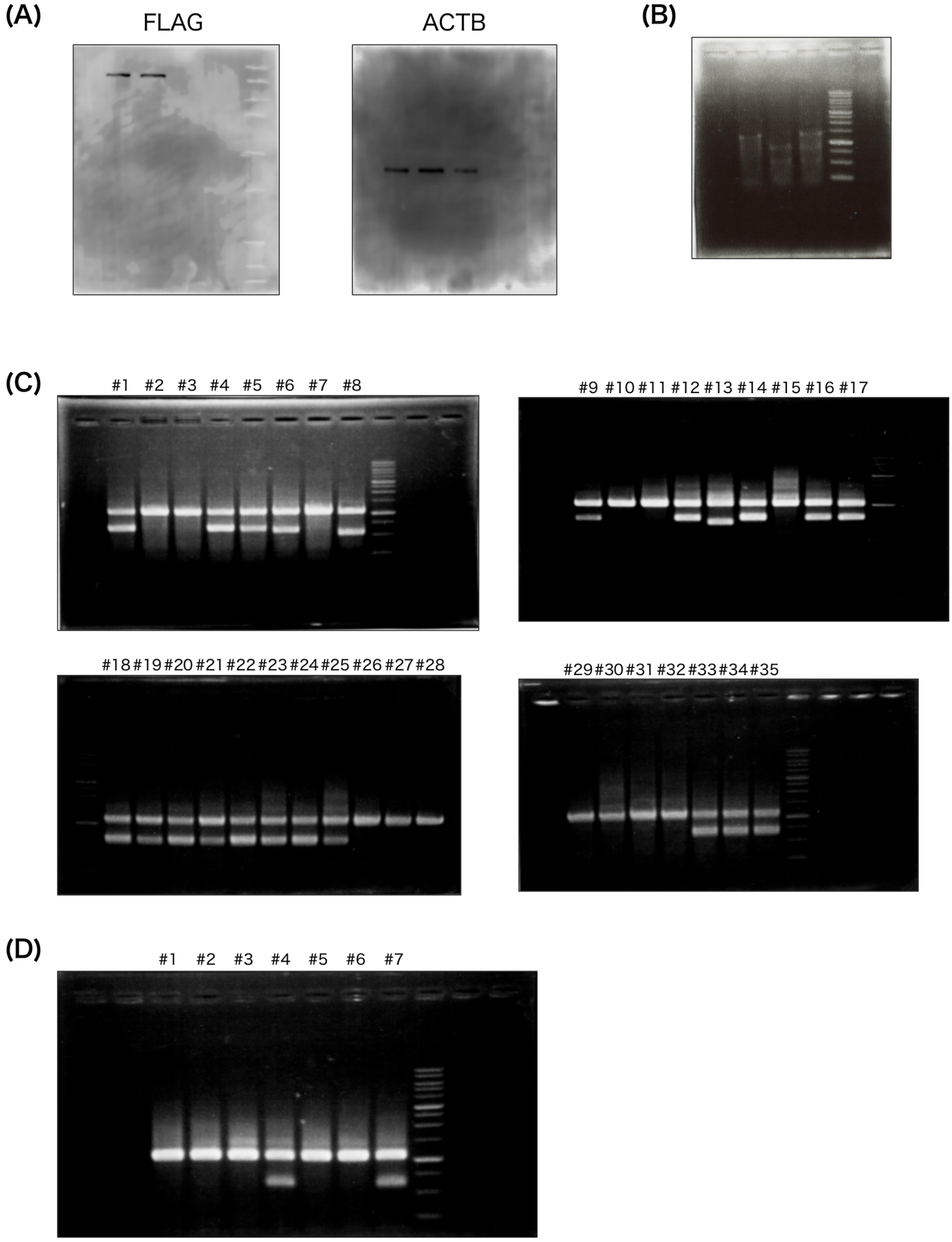

Supplementary Figure 11. Uncropped full-length blot/gel images.

(A) Full-length images of western blotting membrane in Supplementary Figure 1B. (B) Full-length gel image of PCR-RFLP in Supplementary Figure 7B. (C) Full-length gel images of PCR analysis of the *Cdk1* exon 3 deletion in F0 mice in Fig. 3C and Supplementary Figure 9. (D) Full-length gel image of PCR analysis of the *Cdk1* exon 3 deletion in F1 embryos in Fig. 3E.

Supplementary Table 1. Primer sets for genomic PCR.

Target locus	Sequences		
Gt(ROSA)26Sor on-target	Forward	5'-	ACGTTTCCGACTTGAGTTGC
	Reverse	5'-	ATACTCCGAGGCGGATCAC
Gt(ROSA)26Sor off-target	Forward	5'-	AAGGAGGAAGATAATGTTGAGGTTGAGGC
	Reverse	5'-	CTTCCACCTTTCTCTCTTTACTCTGCTC
Tyr target locus 1	Forward	5'-	GCTACAGCCTAGGTAATTTGGAAGG
	Reverse	5'-	CATGTCAACAAAACACCCTTAGACAG
Tyr off-target site 1	Forward	5'-	GGAGTCAGTTCTCCCATCCACC
	Reverse	5'-	CCCTAGCTGTCCTGGAACTCG
Tyr off-target site 2	Forward	5'-	GGGCTGAGGGGACTTATTCTACC
	Reverse	5'-	TTCCTTCCGTCTTCCTTACTTTTTCC
Tyr target locus 2	Forward	5'-	GGGCTATGTACAAACTCCAAG
	Reverse	5'-	TCCATACAAAGAGGTCGTAGATGTTG
Cdx2	Forward	5'-	GTTCCCTGGTTCTGAGGTTCTG
	Reverse	5'-	TTCAGCCAAAGACGAGGGAAG
Cdk1	Forward	5'-	AGTTTCACAGTGCCTCCTCTTG
	Reverse	5'-	TCACCCTGGGTAATTCTGTGAG
