# Supplementary material for: High-fidelity endonuclease variant HypaCas9 facilitates accurate allele-specific gene modification in mouse zygotes
Source: Commun Biol. 2019 Oct 10;2:371. doi: 10.1038/s42003-019-0627-8 (PMC6787007; doi:10.1038/s42003-019-0627-8)
Supplement: Supplementary file 2 — Reporting Summary [file 42003_2019_627_MOESM2_ESM.pdf]

nature research

Corresponding author(s): Wataru Fujii, Ph.D.

Last updated by author(s): Sep 11, 2019

Reporting Summary

Nature Research wishes to improve the reproducibility of the work that we publish. This form provides structure for consistency and transparency in reporting. For further information on Nature Research policies, see Authors & Referees and the Editorial Policy Checklist.

Statistics

For all statistical analyses, confirm that the following items are present in the figure legend, table legend, main text, or Methods section.

n/a	Confirmed	
☐	☒	The exact sample size (*n*) for each experimental group/condition, given as a discrete number and unit of measurement
☐	☒	A statement on whether measurements were taken from distinct samples or whether the same sample was measured repeatedly
☒	☐	The statistical test(s) used AND whether they are one- or two-sided<br>*Only common tests should be described solely by name; describe more complex techniques in the Methods section.*
☒	☐	A description of all covariates tested
☒	☐	A description of any assumptions or corrections, such as tests of normality and adjustment for multiple comparisons
☒	☐	A full description of the statistical parameters including central tendency (e.g. means) or other basic estimates (e.g. regression coefficient) AND variation (e.g. standard deviation) or associated estimates of uncertainty (e.g. confidence intervals)
☒	☐	For null hypothesis testing, the test statistic (e.g. *F*, *t*, *r*) with confidence intervals, effect sizes, degrees of freedom and *P* value noted<br>*Give P values as exact values whenever suitable.*
☒	☐	For Bayesian analysis, information on the choice of priors and Markov chain Monte Carlo settings
☒	☐	For hierarchical and complex designs, identification of the appropriate level for tests and full reporting of outcomes
☒	☐	Estimates of effect sizes (e.g. Cohen's *d*, Pearson's *r*), indicating how they were calculated

Our web collection on statistics for biologists contains articles on many of the points above.

Software and code

Policy information about availability of computer code

Data collection	n/a
Data analysis	The sequence chromatogram data were analyzed with Tracking of Indels by Decomposition (TIDE) version 2.0.1 (https://tide.deskgen.com/), and the sequencing data from HindIII-digested products were analyzed using CRISP-ID version 1.1 (http://crispid.gbiomed.kuleuven.be/).

For manuscripts utilizing custom algorithms or software that are central to the research but not yet described in published literature, software must be made available to editors/reviewers. We strongly encourage code deposition in a community repository (e.g. GitHub). See the Nature Research guidelines for submitting code & software for further information.

Data

Policy information about availability of data

All manuscripts must include a data availability statement. This statement should provide the following information, where applicable:

- Accession codes, unique identifiers, or web links for publicly available datasets
- A list of figures that have associated raw data
- A description of any restrictions on data availability

The sequence information of constructed plasmids were described in Supplemental information and the plasmid DNA have deposited in Addgene (#131467, https://www.addgene.org/Wataru_Fujii/). The data that support the findings are available on request from the corresponding author.

Field-specific reporting

Please select the one below that is the best fit for your research. If you are not sure, read the appropriate sections before making your selection.

☒ Life sciences ☐ Behavioural & social sciences ☐ Ecological, evolutionary & environmental sciences

For a reference copy of the document with all sections, see nature.com/documents/nr-reporting-summary-flat.pdf

Life sciences study design

All studies must disclose on these points even when the disclosure is negative.

Sample size	The sample size was determined based on previous publications (https://www.ncbi.nlm.nih.gov/pubmed/23997119 , https://www.ncbi.nlm.nih.gov/pubmed/29088065).
Data exclusions	No data were extruded from the analyses.
Replication	Biological and technical replicates were performed.
Randomization	Zygotes used for microinjections were randomly picked up from in vitro or naturally fertilized zygotes pools. Sequenced embryos were randomly chosen from the microinjection-derived GFP-positive blastocyst stage-reached embryos.
Blinding	The experimenters were not blinded during the experiments or analysis.

Reporting for specific materials, systems and methods

We require information from authors about some types of materials, experimental systems and methods used in many studies. Here, indicate whether each material, system or method listed is relevant to your study. If you are not sure if a list item applies to your research, read the appropriate section before selecting a response.

Materials & experimental systems

n/a	Involved in the study
☐	☒ Antibodies
☐	☒ Eukaryotic cell lines
☒	☐ Palaeontology
☐	☒ Animals and other organisms
☒	☐ Human research participants
☒	☐ Clinical data

Methods

n/a	Involved in the study
☒	☐ ChIP-seq
☒	☐ Flow cytometry
☒	☐ MRI-based neuroimaging

Antibodies

Antibodies used	anti-Flag M2 monoclonal antibody (F1804, Sigma-Aldrich), anti-β-actin polyclonal antibody (GTX109639, GeneTex, Inc., CA, USA), horseradish peroxidase-conjugated anti-mouse IgG and anti-rabbit IgG (Jackson ImmunoResearch Laboratories, Inc., West Grove, PA, USA)
Validation	Manufacturer's website; (anti-Flag M2) https://www.sigmaaldrich.com/catalog/product/sigma/f1804 , (anti-ACTB) http://www.genetex.com/beta-Actin-antibody-GTX109639.html , (anti-mouse IgG) https://www.jacksonimmuno.com/catalog/products/115-035-003 , (anti-Rabbit IgG) https://www.jacksonimmuno.com/catalog/products/111-035-144<br>Our previous applications; https://www.ncbi.nlm.nih.gov/pubmed/23997119 , https://www.ncbi.nlm.nih.gov/pubmed/29088065

Eukaryotic cell lines

Policy information about cell lines

Cell line source(s)	HEK293 gifted from Dr K Chida of the University of Tokyo (https://www.ncbi.nlm.nih.gov/pubmed/21239526).
Authentication	The cell line used was not authenticated.
Mycoplasma contamination	Not tested.
Commonly misidentified lines (See ICLAC register)	n/a

Animals and other organisms

Policy information about studies involving animals; ARRIVE guidelines recommended for reporting animal research

Laboratory animals	3-week-old female and >8-week-old male C57BL/6Ncr mice (Sankyo Labo Service Corporation, Tokyo), >8-week-old male DBA/2J mice (CLEA Japan, Tokyo), and 8-week-old female ICR mice (Sankyo Labo Service Corporation) were housed at 24 ± 2℃ and 50 ± 10% humidity under a 12/12 h light/dark cycle with free access to water and diet.
Wild animals	This study did not involve wild animals.
Field-collected samples	This study did not involve samples collected from field.
Ethics oversight	All animal care and experimental procedures conformed to the Guidelines for Animal Experiments of The University of Tokyo, and were approved by the Animal Research Committee of The University of Tokyo (approval No. P18-093).

Note that full information on the approval of the study protocol must also be provided in the manuscript.
